# The mechanical behavior dependence on the TiB whisker realignment during hot-working in titanium matrix composites

**DOI:** 10.1038/srep36126

**Published:** 2016-10-26

**Authors:** Fengcang Ma, Ping Liu, Wei Li, Xinkuan Liu, Xiaohong Chen, Ke Zhang, Deng Pan, Weijie Lu

**Affiliations:** 1 School of Materials Science and Engineering, University of Shanghai for science and technology, Shanghai 200093, PR China; 2 State Key Laboratory of Metal Matrix Composites, Shanghai Jiao Tong University, Shanghai 200240, PR China

## Abstract

Low-cost TiB whiskers reinforced titanium matrix composite (TMCs) was fabricated with enhanced mechanical performances using *in situ* technologies and hot working. Morphologies observation indicates that needle-like TiB whiskers with a hexagonal transverse section grow along the [010] direction due to B27 crystal structure and its growth mechanism. Mechanical properties tests show that the mechanical behavior of the TiB whiskers reinforced TMCs is dependent on the deformation amplitudes applied in hot-working. The improvement in yield strength by hot-working is attributed to the TiB whiskers realignment and the refinement of microstructure. Models are constructed to evaluate the realignment of TiB whisker during deformation and the increase in yield strength of the composite at elevated temperatures. These models clarify the alignment effect of TiB whiskers under various deformation amplitudes applied in hot-workings and reveals the yield strength dependence on TiB whiskers orientation.

Titanium matrix composites (TMCs) present high strength at room and moderately elevated temperature. Ceramic reinforcements in TMCs can improve the modulus, strength and creep resistance of the matrix significantly^1^,^2^,^3^. Many reinforcements have been used in TMCs, such as TiB, TiC, SiC, Al_2_O_3_ and TiB_2_. TiB ceramic is regarded as a powerful reinforcement because it presents excellent chemical stability and thermal stability in the Ti matrix^4^,^5^,^6^,^7^,^8^. Among the various designs of TMCs, the *in situ* synthesis techniques, in which reinforcements are formed *in situ* in matrix through reactions between reactants with matrix, have been paid great attention due to the low interface contamination and incompatibility between the matrix and the reinforcements^9^,^10^,^11^,^12^,^13^,^14^,^15^.

The *in situ* processing techniques have evolved over last decades for the preparation of ceramic phase reinforced composites, including self-propagating high-temperature synthesis, mechanical alloying, reactive squeeze casting, exothermic dispersion technology, reactive hot pressing process, reactive spontaneous infiltration, direct reaction synthesis, combustion assisted cast and directed melt oxidation process^16^,^17^,^18^,^19^,^20^,^21^,^22^,^23^,^24^,^25^,^26^. But cost of most preparation methods of TMCs is high. On the other hand, mechanical properties of the TMCs are not improved significantly by the just addition of reinforcement simply. The facile processing is under challenge for the TMCs fabrication with high mechanical properties.

The mechanical behavior of TMCs is different from those of matrix alloy, but it depends on the matrix, the reinforcement, reinforcement/matrix interfaces and processing technology. In recent years, numerous studies have been performed on hot-working to process TMCs based on the traditional hot-working technology of Ti alloys, such as rolling, extrusion and forging. These studies indicate that mechanical properties of the composite are great dependent on the microstructure of matrix, inclusions and second phases, and these factors can be controlled by hot-working^27^,^28^,^29^,^30^,^31^,^32^. Furthermore, Gorsse *et al*.^33^ found that the composite with aligned TiB whiskers along tensile direction presented higher strengths compared to that in which the orientation of TiB whiskers is random. Guo^34^ studied the evolution of TiB whiskers orientation in the composite during rolling suggesting a model to predict the orientation of TiB whiskers with deformation amplitude. However, the effect of TiB orientation on the mechanical behavior of TMCs is not clear enough, and the model between them has not been set up by present studies. And these studies may be very useful in the optimizing mechanical behavior of such composites.

The aim of this work is to study the effect of deformation amplitude applied in hot-working on TiB whiskers orientation and the mechanical behavior dependent on the TiB whisker realignment. In this work, mechanical behaviors of the TMC with various deformation amplitudes applied in hot-working is characterized and analyzed. It was clarified that the improvement in yield strength by hot-working results from the TiB whiskers realignment and the refinement of microstructure. And a model is constructed to evaluate the yield strength of TMCs reinforced with whiskers based on the dependence of the inclinations of whiskers and grain size of matrix.

## Results and Discussion

### TiB whisker morphology and formation mechanism

**Figure 1:**
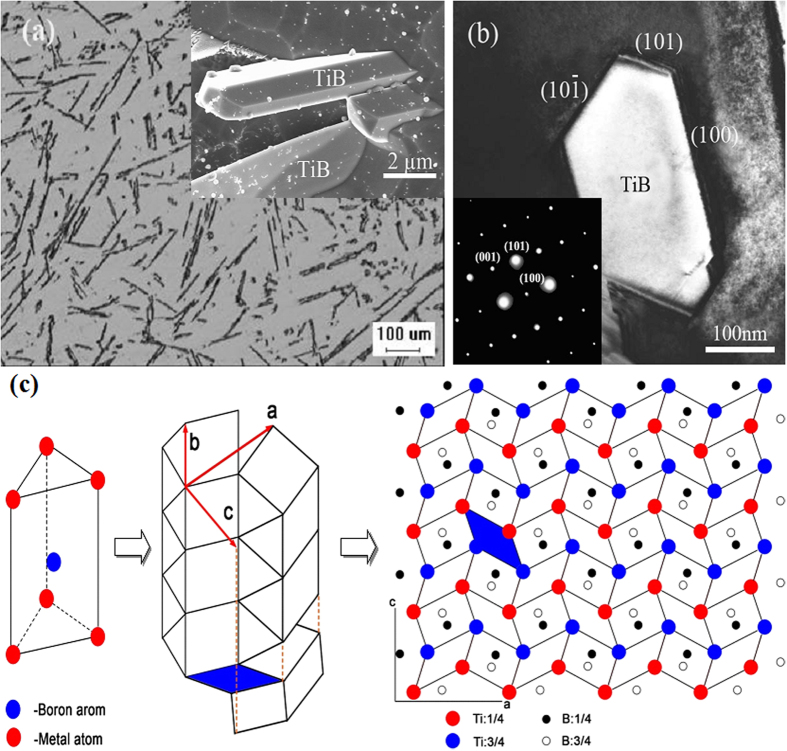
(**a**) Optical morphology and scan electron morphology of TiB, (**b**) Transmission electron microscopy and selected area diffraction patterns of TiB, (**c**) Schematic illustration for the construction of B27 crystal structure of TiB: primitive trigonal prism, connection of trigonal prism, construction of B27 structure and the projection of (010) crystal plane.

Figure 1(a) shows the TiB distribution in the cast composite and morphologies of TiB deep etched. The size of TiB needles diameter in this work are about 1 μm to 3 μm with an aspect ratio about 12.2 ± 4.3. Surface of TiB needles is clean and flat. TEM bright field image of TiB and corresponding selected area diffraction patterns are presented in Fig. 1(b), which indicates that needle-like TiB whisker is B27 orthorhombic structure and lattice parameters *a* = 0.612 nm, *b* = 0.306 nm and *c* = 0.456 nm. And the whisker axis of TiB is parallel to the [010] crystal orientation of the B27 cell. TiB grow faster along the [010] direction leading to a rod-like morphology that was reported by several research groups. Li *et al*.^35^ found that TiB prepared using the reactive hot pressing was elongated needles with hexagonal cross-sections. The size of TiB whiskers in cross-section is about 1~2 μm with an aspect ratio about 20. Rod-like TiB with a size 2~6 μm in cross-section and an aspect ratio 10~15 was prepared in combustion assisted cast processed Ti matrix composite by Ranganath *et al*.^36^. Similarly, rod-like TiB with a length about 4 μm and aspect ratios 5~10 was also prepared in reactive squeeze casting processed TMCs^37^,^38^,^39^,^40^. Diameter and aspect ratio of TiB whiskers may be different through various synthetic methods. The diameter of TiB in this work is similar to that of TiB by reactive hot pressing, but its aspect ratio is similar to that using CAC method.

The formation of needle TiB morphology can be explained from its crystal structure. Lundstrom^41^ suggested the crystal structures of many borides are all constructed by the same crystal cell: a trigonal prism with a boron atom lying at the center and six Ti atoms lying at six vertexes. In the B27 crystal structure, such trigonal prisms are assembled along their edges. Along the [010] direction of B27 structure, boron atoms form zigzagging chains and boron-free ‘pipes’ with a trapezoidal cross-section are formed by Ti atoms, as presented in Fig. 1(c). According to the ‘Periodic Bond Chain’ theory, TiB would grow faster along the [010] direction of B27 than along the [10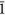], [101] and [100] directions. And TiB would grow into needle or whisker morphology with the axis parallel to the [010] crystal direction of B27 structure. Because growth of crystals is usually restricted by the slowest-growing crystal planes, (10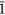), (101 and (100) planes form the surfaces of TiB whiskers, as presented in Fig. 1(b,c).

### Modeling of the realignment of TiB whiskers by hot-working

**Figure 2:**
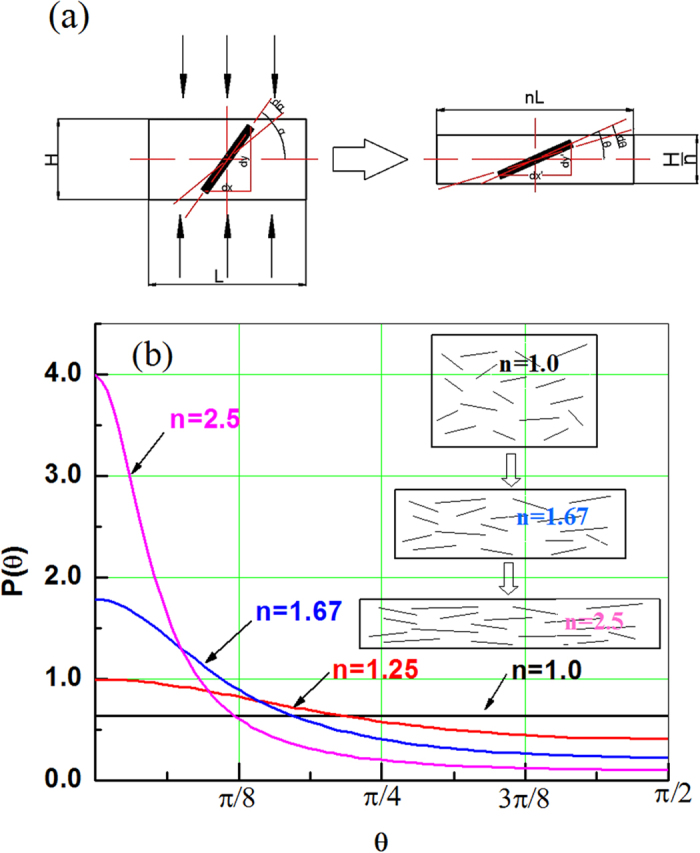
(**a**) Model for whisker orientation as a function of deformation amplitude, (**b**) TiB whisker orientation probability density function variation with the deformation amplitude applied in hot-working.

It is well understood that TiB whiskers in the composite realign along the flow direction during composite deformation. Guo^21^ studied the realignment effect of TiB by rolling suggesting a model to evaluate the variation of TiB whiskers orientation with deformation amplitude. In this model, the orientation of TiB whiskers is defined as the orientation angle that is the angle of length directions of TiB with respect to the rolling direction, and the variation of orientation angle of TiB whiskers with deformation amplitude is described by a probability density function. In Eq. (1), P(α) is the initial probability density function, but P(θ) is the probability density function after the hot-working, as presented in Fig. 2(a), then the following equation can be obtained:


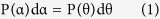


If the thickness of the composite becomes 1/n of the initial one after the hot-working, the of TiB whisker orientation (orientation angle θ after deformation) can be calculated with the initial orientation angle α and the deformation amplitude (n), as presented in Fig. 2(a). The orientation angle θ after deformation can be calculated with Eq. (2):


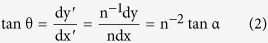



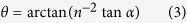


In Eq. 1–3 α is the initial orientation angle of TiB whisker (the angle of the length of TiB whisker direction respect to horizontal direction) and θ is the orientation angle of the TiB whisker after deformation.

Derivation of Eq. (3) to α:


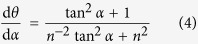


Substituting tan *α* = *n*^2^tan *θ*, which is based on the Eq. (2), into Eq. (4):


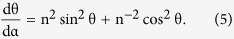


Substituting Eq. (5) into Eq. (1):


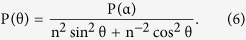


Due to the initial orientation angles of TiB whiskers distributing uniformly from 0 to 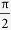 in the cast composite, the probability density of TiB orientation angles is a constant. Because the integral value of the probability density from 0 to 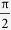 is 1, then


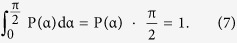


Thus,


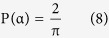


Substituting this value into Eq. (6):


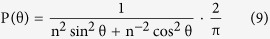


According to Eq. (9), the probability density function of TiB whiskers P(θ) is a function of the deformation amount. Figure 2(b) shows the effect of the deformation amplitude applied in this work on the probability density function P(θ). As presented in Fig. 2(b), the increase of deformation amplitude applied in hot-working decreases the orientation angle of TiB whiskers significantly, which should affect the mechanical behavior of the composite.

### Modeling of strengthening effect of TiB whisker

A shear-lag model^42^ was used to evaluate the strengthening effect of TiB whiskers in composites with the length direction of whiskers parallel to the tensile direction.


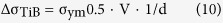


where σ_ym_ is the yield strength of the matrix. l/d and V are the aspect ratio and the volume fraction of the whiskers, respectively. In Eq. (10), the length direction of the whiskers must be parallel to the loading direction during the tensile tests of the composite. However, the orientation of TiB whiskers in this composite does not meet this requirement. The orientation of the TiB whisker in the composite on its strengthening effect should be taken into account.

For such composites in which orientation of TiB whiskers is disorder in the matrix, Fukuda *et al*.^43^ suggested a probability theory to evaluate orientation and length of whiskers on its strengthening effect in composites reinforced by whiskers. In this model, the yield strength increase of the composite by TiB (Δσ_TiB_) was modified by adding an strengthening factor C_0_ ranging from 0 to 1 in Eq. (10), and C_0_ is dependent on the orientation and length of TiB whiskers in the composite. The strengthening effect of TiB can be predicted by Eq. (11), and C_0_ is given by Eq. (12):


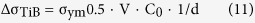



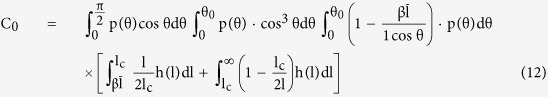


where θ is the orientation angle of TiB, i.e. the angle of the length direction of whiskers with respect to tensile direction; θ_0_ is the critical angle that determines whether a whisker is a “bridging fiber”or not; p(θ) is the TiB orientation angle probability density function; h(l) is the TiB whiskers length probability density function; β is a constant ranging from 0 to 1 determining whether a whisker is a “bridging fiber” or not^44^; l, lc and 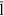, are the maximum length, critical length and average length of the TiB whiskers, respectively. The value of average aspect ratio was calculated to be 12.2 ± 4.3, but that of the critical aspect ratio of TiB whiskers is about 2.07 45. Due to the significantly higher value of average aspect ratio compared with that of the critical aspect ratio, all TiB whiskers in the present composite are regarded as the bridging whiskers in order to simplify the model. Then β is equal to 0 and θ_0_ is equal to 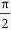. Under above assumption, based on Eq. (12) C_0_ can be simplified to be:


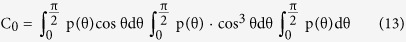


Based on Eq. (13), it is necessary to evaluate the probability density function of TiB whiskers (p(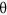)) firstly in order to calculate its strengthening factor C_0_.

It is well understood that TiB whiskers formed randomly in the preparation process of the composite. Then, TiB whiskers orientation angles in the cast composite distribute uniformly from 0 to 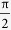, and the TiB whisker orientation angles probability density is a constant. Because the integral of this probability density from 0 to 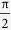 is 1.


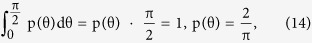



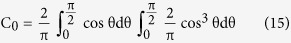


Substituting Eq. (9) into Eq. (13), TiB whisker strengthening factor variation with deformation amplitude applied in hot-working on can be calculated by Eq. (16):


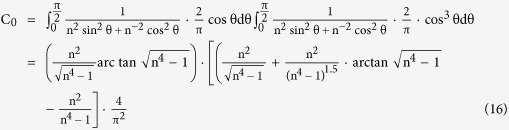


### Effect of TiB whiskers realignment on mechanical behavior of TMC

**Figure 3:**
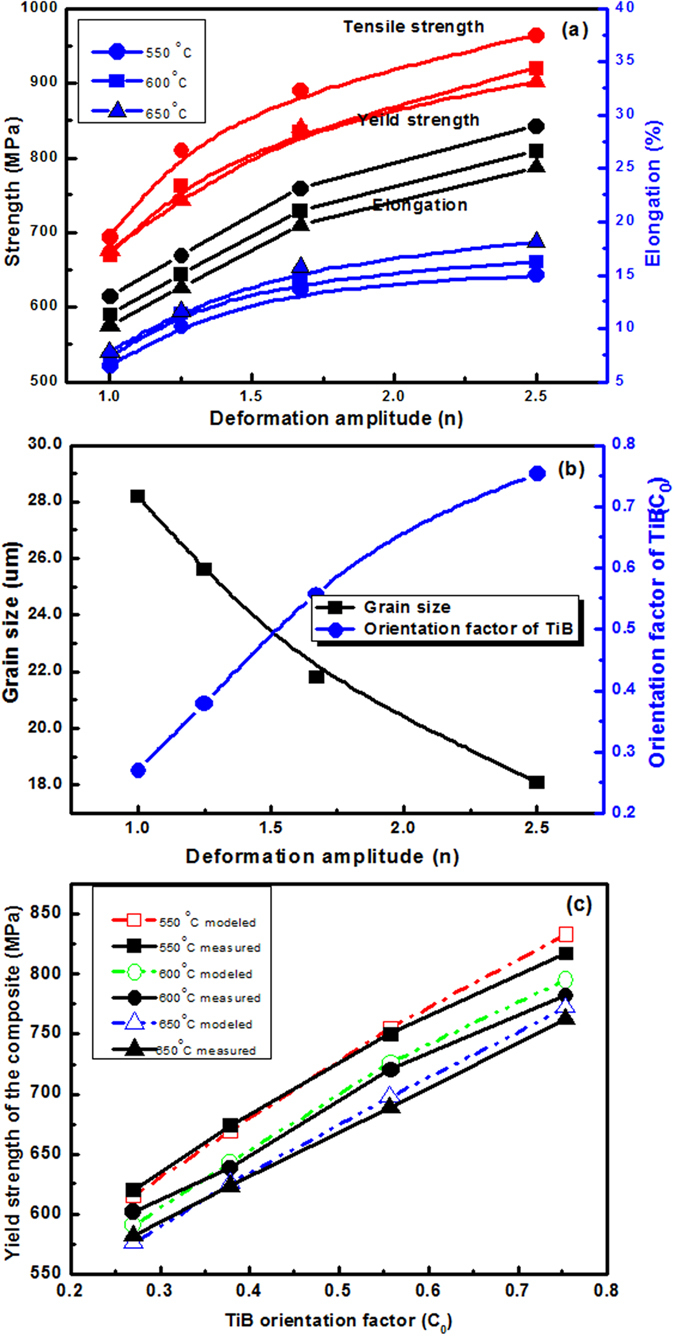
(**a**) Tensile properties of the TMC at different test temperatures, (**b**) Evolution of grain size (Gd) and TiB whiskers orientation factor (C_0_) with deformation amplitude, (**c**) Modeling of the yield strengths of the composite versus TiB orientation factor (C_0_).

Mechanical properties of the composite with various deformation amplitudes by tensile tests are presented in Fig. 3(a), in which the tensile direction is parallel to the longitudinal direction of the composite rods. Figure 3(a) shows that hot working increases yield strength of the composite significantly. The increase of the yield strength by hot-working may result from two factors: (a) TiB whisker realignment by deformation of the composite. (b) Grain/microstructure refinement because of dynamic recrystallization during the hot-working. Figure 3(b) shows the effect of TiB whisker realignment and grain/microstructure refinement of the composite by the hot working with various deformation amplitudes.

The increase of yield strength of the composite by grain/microstructure refinement can be calculated by Hall–Petch equation^46^. The strengthening effect by grain/microstructure refinement is not ignorable when the volume fraction of reinforcement in composite is low^47^. The strengthening effect by the grain/microstructure refinement can be calculated by:


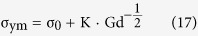


where σ_ym_ and σ_0_ are the yield strength and strength constant of the matrix, respectively. K and Gd are the strengthening coefficient and the grain diameter, respectively^48^. As Eq. (11) described, yield strength of the composite can be modeled as following including the TiB whiskers strengthening effect:


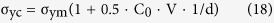


In above equation, σ_yc_ represents the composite yield strength. Taking account into the strengthening effect by grain/microstructure refinement, substitute Eq. (17) into Eq. (18):


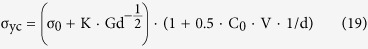


Figure 3(c) presents the modeled yield strengths with Eq. 19 and the measured values of the composite by tensile tests. As presented in Fig. 3(c), the modeled and measured results are in good agreement with each other and show that this model is effective. But for the composite with large amplitude deformation, all yield strengths modeled by this model are larger slightly than measure values. The overestimation in yield strength for the composite with large amplitude deformation may result from the break of some TiB whiskers during hot-working, which is a common observation in the composite with large amplitude deformation. The break of TiB whisker decreases its strengthening effect due to the decrease in its aspect ratio, but this effect has not been included in this modeling.

Based on above results and discussion, it can be seen that the mechanical behavior of the TMCs is dependent on orientation of TiB whiskers. The improvement in yield strength by hot-working can be attributed to the TiB whiskers realignment and the refinement of grain/microstructure. A suggested model can be used to evaluate the yield strength of TMCs including the realignment of TiB whisker with various deformation amplitudes applied in hot-working.

## Methods

### Preparation of the *in situ* composite

**Figure 4:**
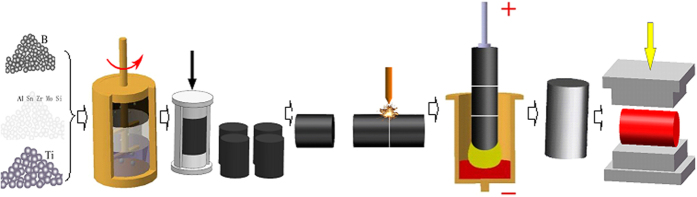
Preparation flow chart for the composite.

Composite used in this work was TiB ceramic reinforced Ti-1100 matrix. The composite preparation flow chart is presented in Fig. 4. In the preparation of this composite, the raw materials are sponge titanium (grade I), boron powder (99.95%, particle size 4~8 μm), aluminum thread (98%), silicon powder (99.95%, particle size 6~12 μm), sponge zirconium (99.5%), and Ti–Sn, Al–Mo master alloys. The composition of Ti-1100 was prepared with Ti–6Al–2.75Sn–4Zr–0.4Mo–0.45Si in weight percent. Sponge titanium, boron powder and other raw materials were prepared with 4 v. % TiB/Ti-1100 component, and volume fraction of the TiB was added with formula (20).


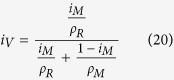


In above formula, *i*_*V*_, *i*_*M*_, *ρ*_*R*_ and *ρ*_*M*_ is TiB volume fraction, TiB weight fraction, density of TiB and density of Ti-1100 alloy, respectively.

Proportional sponge titanium, boron powder and other raw materials were mechanically mixed firstly. Then, the mixed raw materials were pressed into preformed bars in a mold with a press machine. The preformed bars were welded into big ones as the electrode used in the subsequent melting processing. The electrode bars was melted in a vacuum arc furnace. During the melting process, through the reaction between titanium with boron TiB was prepared (Ti + B → TiB). In order to improve the composite chemical homogeneity, the composite was melted three times. Homogenization of the composite was carried out at 1423 K for 1.5 h then was cooled by air. After homogenization, the composite was treated by a hot working on a pressure machine. Before the hot-working, molds used in the hot working were heated to 1123 K, the composite was heated to 1273 K. The composite temperature measured by infrared device was ensured above 1123 K during the hot-working treatment. After hot-working, samples were cooled to ambient temperature by water. The deformation amplitude applied in the hot-working was calculated with the reduction ratio in thickness, where 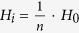, *H*_0_ is the thickness of the sample before hot-working, and *H*_*i*_ is the thickness of the sample after hot-working. In particular, it should be pointed out that the composite preparation method used in this work was same to the preparation method that usually used to produce Ti alloys, as presented in Fig. 4.

### Characterization

Morphology observation samples of the composite were cut from ingot then ground and mechanical polished. Samples for scan electron microscopy (SEM) observation were etched by Kroll’s reagent (2~6 mL HF, 4~12 mL HNO_3_, 100 mL water). Samples for transmission electron microscopy (TEM) observation were prepared with a twin-jet device operated at 293~303 K and 30~50 V with an electrolyte 10% HClO_4_ and 90% CH_3_COOH. TEM samples were observed on a JEM-2010 electron microscope operated at 200 kV.

Plate samples for tensile tests were machined from the composite with 3.0 mm in thickness, 12 mm in width and 50.0 mm in gauge length. Tensile tests were carried out on a Zwick/Roell 100 kN tensile testing machine. Before tests, the tensile samples were heated to test temperatures in a furnace equipped on the tensile machine and held for 5 min. The tensile tests started with 5.0 × 10^−3^ s^−1^ strain rate. Tensile tests were carried out three times for each sample, and the presented result of the sample was the average of the three times.

## Additional Information

**How to cite this article**: Ma, F. *et al*. The mechanical behavior dependence on the TiB whisker realignment during hot-working in titanium matrix composites. *Sci. Rep*. **6**, 36126; doi: 10.1038/srep36126 (2016).

**Publisher’s note**: Springer Nature remains neutral with regard to jurisdictional claims in published maps and institutional affiliations.
